# Challenges of Post-measurement Histology for the Dielectric Characterisation of Heterogeneous Biological Tissues

**DOI:** 10.3390/s20113290

**Published:** 2020-06-09

**Authors:** Alessandra La Gioia, Martin O’Halloran, Emily Porter

**Affiliations:** 1 Translational Medical Device Laboratory, National University of Ireland Galway, Costello Road, H91 TK33 Galway, Ireland; martin.ohalloran@nuigalway.ie; 2 Department of Electrical and Computer Engineering, The University of Texas at Austin, Austin, TX 78712, USA; emily.e.porter@ieee.org

**Keywords:** dielectric measurements, biological tissues, open-ended coaxial probe technique, histology

## Abstract

The dielectric properties of biological tissues are typically measured using the open-ended coaxial probe technique, which is based on the assumption that the tissue sample is homogeneous. Therefore, for heterogeneous tissue samples, additional post-measurement sample processing is conducted. Specifically, post-measurement histological analysis may be performed in order to associate the measured dielectric properties with the tissue types present in a heterogeneous sample. Accurate post-measurement histological analysis enables identification of the constituent tissue types that contributed to the measured dielectric properties, and their relative distributions. There is no standard protocol for conducting post-measurement histological analysis, which leads to high numbers of excluded tissue samples and inconsistencies in the resulting reported data for heterogeneous tissues. To this extent, this study examines the post-measurement histological process and the challenges in associating the acquired dielectric properties with the different tissue types present in heterogeneous samples. The results demonstrate that the histological process inevitably alters the morphology of samples, thus introducing errors in the interpretation of the dielectric properties acquired from heterogeneous biological samples. Notably, sample size was seen to shrink by up to 90% through the histological process, meaning that sensing volume determined from fresh tissues is not directly applicable to histology images.

## 1. Introduction

Accurate dielectric measurements of biological tissues are crucial for the development of electromagnetic diagnostic and therapeutic devices, such as microwave breast imaging systems and microwave ablation applicators [1,2,3]. These technologies are designed based on estimates of the dielectric properties of diseased and healthy tissues. Dielectric properties of biological tissues are typically measured in the microwave frequency range using the open-ended coaxial probe technique [4,5,6,7]. This technique is preferred over others, such as the transmission line method or the cavity perturbation method, because it is nondestructive (it does not require specific sample shape or size) and can be used for in vivo measurements [8,9]. The dielectric measurement procedure with an open-ended coaxial probe is straightforward; however, several factors can introduce uncertainties into the dielectric data, which can consequently reduce the efficacy of electromagnetic medical technologies [9,10]. Generally, uncertainties are higher in dielectric measurements of heterogeneous tissue samples [4] due to the fact that the open-ended coaxial probe technique is based on the assumption that the measured sample is homogeneous [11].

Post-measurement histology has been conducted to associate the dielectric properties acquired by the measurement probe with the different tissue types within heterogeneous samples [12,13,14]. Histology is used by pathologists worldwide to support diagnosis of diseases at a rate of millions of samples per day [15]. Despite this extremely frequent usage in hospitals, the histological process was not designed to support interpretation of dielectric measurements. In fact, histology has been used only recently to associate the measured dielectric properties to the different tissues within heterogeneous tissue samples. As such, there are no standard methods used across the literature for accurately interpreting the relationship between the dielectric properties of heterogeneous tissue samples and the histology of these samples [9]. A key difficulty in linking the dielectric measurements to the histological results is the need for the identification of a “histology region”, i.e., a region demarcated on the histological images that includes the tissues that may have contributed to the measured dielectric properties. The definition of the histology region is challenging because it requires knowledge of the sensing volume of the measurement probe, which is delineated by sensing radius and sensing depth [16,17]. 

While recent studies have investigated the sensing radius and sensing depth of several coaxial probes [16,17], there are no studies in the literature that have investigated the post-measurement histology process and its impact on the definition of the histology region. Furthermore, in all dielectric studies investigating the dielectric properties of heterogeneous tissue samples with histology to date, the histology region has been defined by directly applying the sensing volume findings without taking into account the change in tissue morphology caused by the histological process [18].

In general, studies have reported high numbers of tissue samples that had to be excluded from further analysis due to challenges with the histology procedure including lack of a clear marker of the dielectric measurement location and tissue deformations [13]. For example, in two of the largest studies to date where histology was used to investigate the dielectric properties of breast tissues, more than 24% [13] and 49% [12] of samples, respectively, had to be excluded from further analysis due to histological challenges. Such substantial numbers of excluded samples are particularly unfortunate when human samples are obtained yet not able to be fully analysed. Furthermore, even with samples that can be analysed following histology, the questions discussed above related to what histology region to use in order to accurately interpret the histology in the context of the dielectric measurement are still an issue.

For these reasons, this study investigates the process of conducting post-measurement histological analysis. This work aims to provide a concrete understanding of how the histology process impacts tissue samples in the context of associating dielectric properties to the samples, and specifically examines the challenges of identifying the histology region even when the sensing volume is known. In particular, this study involves dielectric measurement and post-measurement histology of heterogeneous tissue samples from different animals and different regions of the body. Furthermore, in this study, the sensing radius and tissue dielectric contribution findings from previous dielectric studies [17,19,20] are considered in order to evaluate the applicability of these findings in defining the histology region and in accurately interpreting the measured dielectric properties. The specific findings of each of these studies is discussed in the relevant sections below in relation to the results of this work.

The dielectric and histology protocols adopted for the dielectric characterisation of the biological tissue samples are provided in Section 2. In Section 3, after highlighting the challenges and limitations of histology, the results from the histological analysis are reported and associated with the measured dielectric data. Lastly, the key findings of this study are summarised in Section 4.

## 2. Materials and Methods

This section describes the methodology used to examine the post-measurement process and its impact on the dielectric characterisation of heterogeneous biological samples. Specifically, the methodology consists in dielectrically characterising a set of radially heterogeneous biological samples with the support of post-measurement histology. To this extent, Section 2.1 motivates the choice of the radially heterogeneous tissue samples, Section 2.2 details the dielectric measurement protocol, and Section 2.3 describes the histology protocol.

### 2.1. Selection of Radially Heterogeneous Tissue Samples

Biological samples presenting simple radial heterogeneities, such as kidney and muscle with fat inclusions, were selected. Radially heterogeneous samples were chosen because, as opposed to complex heterogeneous samples, it is straightforward to identify a “landmark” location on these tissue sample, which can be marked and tracked from the time of the dielectric measurement through to the post-histology images. Specifically, the landmark location that can be marked is the interface between tissue types. Similarly to the samples analysed in [17,19,20], each of the samples has a structure that does not vary with depth and consists of two tissue types, which have an interface parallel to the axis of the probe and perpendicular to the surface of the sample. In particular, ten samples were selected as representative radially heterogeneous samples to be analysed by post-measurement histology. The ten samples consisted of the following: three samples of ovine kidney (composed of medulla and cortex tissue), two samples of ovine muscle (with fat inclusions), and five samples of porcine muscle (with fat inclusions). All of the samples were obtained from a local butcher, and each sample is from a different source animal. 

**Figure 1:**
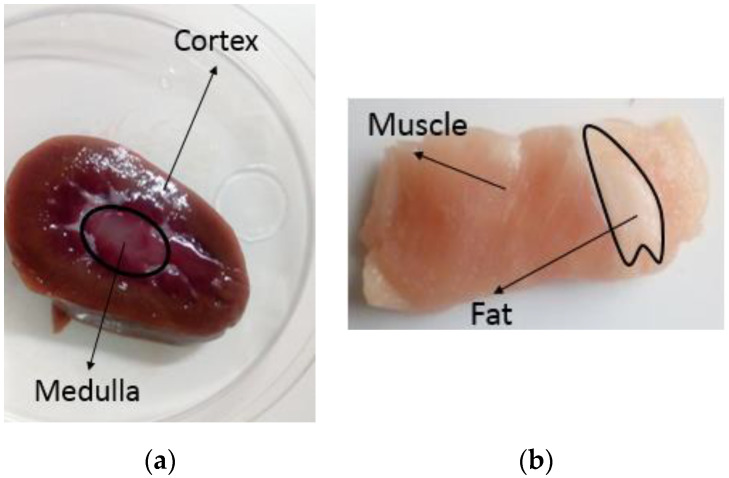
Examples of selected radially heterogeneous samples: (**a**) ovine kidney sample, where the medulla region is easily distinguishable from the cortex region; (**b**) porcine muscle sample, where the fat tissue is easily distinguishable from the muscle tissue. These photos are cross-sections through the longitudinal (xy) plane.

While the three ovine kidney samples had consistent structures with defined cortex and medulla regions, like the sample illustrated in Figure 1a, the two ovine and the five porcine muscle samples had structures that were different from each other. An example of the heterogeneity of ovine and porcine muscle samples is reported in Figure 1b.

### 2.2. Dielectric Measurement Protocol

**Figure 2:**
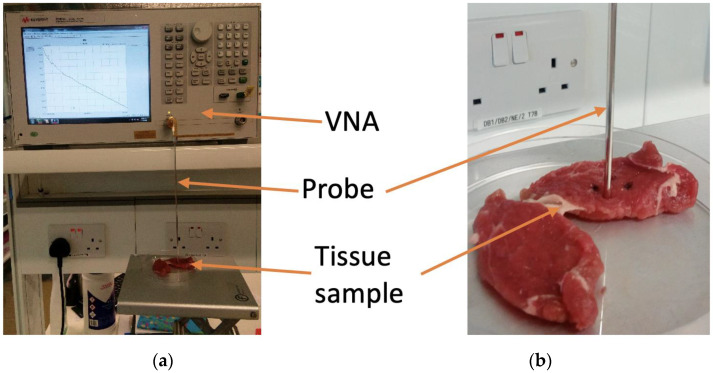
(**a**) Photograph of the dielectric measurement set-up, with Vector Network Analyser (VNA), probe, and tissue sample labelled. (**b**) A zoomed-in version of the photograph illustrates the contact of the probe with the sample.

Dielectric experiments were performed with the Keysight slim form probe connected to the Agilent E8362B Vector Network Analyser (VNA) across the MW range of 2–6 GHz, which is the operating frequency of many MW imaging systems and MW ablation applicators [2,3]. The slim form probe was selected for consistency with the experiments detailed in [17,19,20], the findings of which were used to support the association of the measured dielectric data with the histology of the measurement samples. Specifically, the findings from [19,20], which quantitatively examined the sensing radius of the Keysight slim form probe, the most commonly used probe in recent studies [5,6,9], were used to define the histology region. The findings from [17,19] were also considered for the estimation of the dielectric contribution of each constituent tissue to the measured dielectric data. A photograph of the experimental set-up showing the slim form probe connected to the VNA is provided in Figure 2.

For each dielectric experiment, data were collected at 21 frequency points. Furthermore, the same measurement procedure was followed as in [20]. Before each measurement set, the system was calibrated and validated. The validation involved measurements of the known, well-characterised material 0.1 M NaCl, after the calibration of the system, and before and after each tissue measurement. The temperature of the 0.1 M NaCl solution was recorded at each measurement instance in order to compare the measured data with the known dielectric properties of 0.1 M NaCl at that temperature. Throughout the measurement process, it was ensured that the measurement uncertainty was consistently within the total combined uncertainty reported in [20], i.e., 2.5% for relative permittivity and 4.2% for conductivity. 

Prior to measurements, tissue samples were kept in cling film to minimise tissue dehydration by limiting the exposure of each sample to air. During tissue measurements, each sample was brought to the probe tip using a lift table, a firm contact between the probe and the tissue was kept after finding the optimal probe–tissue pressure, excess blood on the surface of the sample was removed using cotton swabs, and the tissue temperature, which was consistently between 21 and 22 °C, was measured with an infrared thermometer. The optimal probe–tissue pressure resulting in consistently repeatable dielectric measurements was found by performing multiple measurements at the same location with a gradually increased pressure. Thus, the optimal probe–tissue pressure for a specific tissue location was the one at which the measured dielectric properties had approximately the same magnitude. Furthermore, between each measurement, the probe was cleaned with an alcohol wipe in order to avoid contamination.

Dielectric experiments were conducted on the samples listed in the previous subsection. Specifically, measurements conducted on ovine kidney covered three regions of the sample: the homogeneous cortex region, the homogeneous medulla region and the radially heterogeneous region at the cortex–medulla interface. Measurements conducted on porcine and ovine muscle covered multiple regions of the sample, consisting of homogeneous muscle, homogeneous fat, radially heterogeneous regions with side-by-side or concentric muscle and fat. 

After conducting the dielectric measurements, the measurement sites were marked with a histology marker and then prepared for sample processing. The samples were then subject to the histology steps detailed in the following subsection. It is noted that all dielectric measurements were conducted prior to the sample being preserved and processed for histology, as these procedures are expected to change the dielectric properties of the tissue sample [21].

### 2.3. Histology Protocol 

The protocol designed for post-measurement histology consists of six steps: fixation, processing, embedding, slicing, staining and imaging. The steps followed in this work are the same as those used in standard clinical or research histology workups, and have not been altered or adapted for the purpose of this study.

Labelling was essential to ensure that the samples and measurement sites could be uniquely identified throughout each step of the histology protocol. Furthermore, since parts of the histological process can cause shrinkage and deformation of the tissue samples [15,18], a subset of five samples were measured with a calliper, before and after the two steps of fixation and processing, in order to monitor the change in size of the samples. Monitoring of the change in size of the sample supports identification of the measurement sites on the histology slides in order to find an accurate correspondence between measured dielectric data and histological content. 

To preserve the histology of the tissue sample, fixation was conducted by immersing the sample into a 10% ***w/v*** formalin solution. In order to guarantee adequate staining samples were kept in formalin solution from 48 h to 12 days [15], after measurement but before tissue processing.

**Figure 3:**
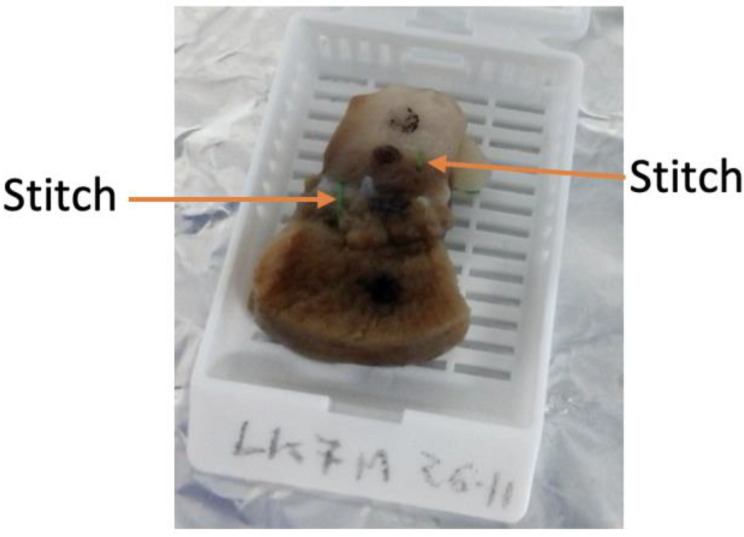
Ovine kidney sample after undergoing fixation in 10% *w/v* formalin solution. Two green stitches can be seen beside the marked measurement sites across the heterogeneous regions of the sample. The sample is placed into a cassette to be inserted into the tissue processor. The cassette was labelled with the unique identifier, i.e., LK7M 26-11, written in pencil since graphite is resistant to all chemicals used by the tissue processor.

Either before or after fixation, the samples were sectioned in order to be fitted into the cassettes used by the Thermo Scientific Excelsior tissue processor. Furthermore, preliminary histology experiments showed that the black marks on samples with a glossy texture (like kidney) were not resistant to sample processing. For this reason, in order to avoid exclusion of the samples (as occurred in [12,13]), the measurement sites across heterogeneous sample regions of the kidney samples were further marked with stitches. An example of the stitches being used for marking is reported in Figure 3. Specifically, two green stitches can be observed beside the measurement sites; they were not placed over the measurement sites in order to avoid tissue damage. The distances between the stitches and the marked measurement sites were recorded at this stage and also after tissue processing.

After fixation, the samples were placed into cassettes, the tissue processor was run, and they were embedded in paraffin wax. The orientation of each sample was taken into account during embedding, so that the measurement sites on each sample were easily identifiable. Samples were then sliced with a microtome and placed on slides. The slice thickness was generally 5 μm, which allows for clear images of the slice as it contains only a single layer of cells. The slides were labelled to keep track of the sequence of the slices, i.e., to facilitate the association of each slice to a specific depth of the sample. Next, haematoxylin and eosin (‘H&E’) staining was performed as reported in [22]. After staining, the slides were mounted using the Dibutylphthalate Polystyrene Xylene (DPX) mounting medium between the slide and the coverslip, and left to dry for a day at room temperature. Finally, the slides were imaged with the Olympus VS120 digital slide scanner using from 50 to 100 focal points for each slice. The slides were analysed with Olyvia (version 2.9.1) and Image J. The results of the histological analysis are discussed in the following section.

## 3. Results and Discussion

This section discusses the correspondence between measured dielectric data and sample tissue content as identified through histology. Specifically, Section 3.1 reports the challenges of the histology process and how, despite best efforts, these challenges brought about the exclusion of four out of ten samples. Then, Section 3.2 reports the analysis performed to associate the dielectric data with the sample histological content. 

### 3.1. Histological Analysis and Challenges 

This subsection overviews each step of the post-measurement histology process conducted on the heterogeneous samples. 

Firstly, fixation was performed. Different fixation times can have different impacts on the quality of the staining and therefore on the quality of the images at the microscope [15]. However, among the ten samples that underwent different fixation times (from 2 to 12 days), no notable difference in staining quality was seen for the purposes of tissue content assessment. Thus, all samples could be analysed in order to differentiate the different tissue content regions. However, it is important to note that the histological assessment was completed visually. For computer-automated methods, precision in the quality of staining may be more important. 

Next, tissue processing was performed. A considerable difference in sample size was found across the steps from fixation to sample processing (prior to embedding). The change in size was monitored for five samples and is reported in Table 1. Among those five samples, two samples (Samples LK1 and LK2) were ovine kidney and three samples (Samples PM1, PM2 and PM3) were porcine muscle. As is clear from Table 1, the change in size for the ovine kidney was generally higher than for the porcine muscle. However, for both sample types, a general small increase in size was recorded between prefixation and postfixation (similarly to what was found for ocular tissues in [23]) and a general decrease in size was recorded between postfixation and postprocessing (due to dehydration). 

Furthermore, from Table 1, it can be noted that the change in size is not consistent across samples, but it depends on the sample constituent tissues and initial shape [18,23]. For example, Samples LK1, LK2 and PM2 exhibited from 18% to 25% postprocessing shrinkage in one direction. Furthermore, a change in size of up to 22% was recorded for Sample PM3, due to the high fat content. In fact, it was noted that fat tissue tended to shrink and harden more than muscle tissue in the tissue processor.

After removing the samples from the tissue processor, it was found that four samples were damaged either entirely or only on the surface during the sample processing. This sample damage can be attributed to the fact that the sample sizes were modified through the processing, resulting in compression against the cassettes. At this stage, only one sample was excluded, while the other three samples were further processed, since the damage of these samples involved only the surface.

During embedding, care was taken to put the appropriate amount of paraffin wax so that the sample was moulded firmly into the cassette and the formation of air bubbles was prevented (in order to facilitate sample slicing).

After the samples were embedded, the samples were sliced using the microtome. The slicing of the samples was challenging for fibrous tissue samples (e.g., muscle samples). In fact, the tissues from fibrous samples tended to tear and crumble during the slicing. At this stage, three ovine and porcine muscle samples were excluded. Furthermore, two ovine kidney samples were fibrous only across the first 0.1–0.5 mm. However, the ovine kidney samples were not excluded from the analysis, since the samples were heterogeneous only radially and thus the surface histological content did not contain additional information. 

Through this section, it has become clear why studies such as [12,13] end up with a high rate of sample exclusion. Up to this stage, four out of the ten samples had to be excluded, thus leaving six samples for further analysis. For each of the remaining six samples that underwent histology, five to ten slides were analysed each in order to verify that the samples were heterogeneous only across the radial extent (and not across the longitudinal extent). Hence, a subset of slices were imaged by the digital scanner and analysed to associate the measured dielectric data to the sample histology content.

### 3.2. Dielectric Characterisation of Radially Heterogeneous Samples through Histology

**Figure 4:**
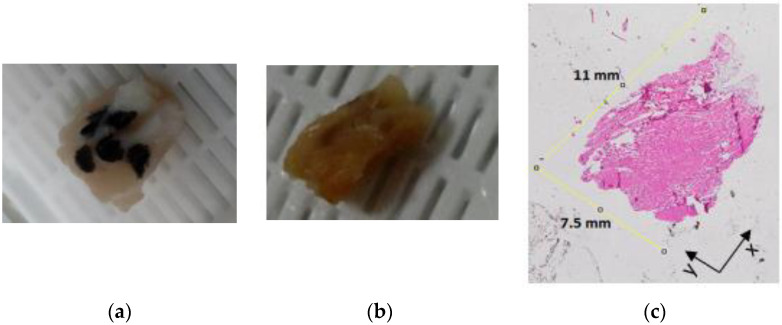
(**a**) Postfixation picture, (**b**) postprocessing picture and (**c**) 5 μm thick histological slice of Sample PM1 (reported in Table 1). The five marked measurement sites in (**a**) are faded in (**b**). Among the five measurement sites, one is from muscle, two from fat (from smaller and larger regions), and two from concentrically heterogeneous regions.

Figure 4 shows the histology of Sample PM1 reported in Table 1. In order to facilitate the association of the dielectric data with the histological tissue content, postfixation and postprocessing sample pictures are reported together with a sample slice imaged with the microscope. Figure 4a presents the five marked measurement sites, which are still visible (although faded) in Figure 4b. Among the five measurement sites, one was from muscle, two from fat, and the remaining two from two concentrically heterogeneous regions consisting of muscle or fat (inner tissue) entirely surrounded by fat or muscle (outer tissue). The two fat measurement sites are from a smaller region (approximately as large as the probe) and a larger region (twice larger than the probe), which were considered for the validation of the sensing radius findings in [19,20]. Furthermore, Figure 4c reports the 5 μm slice obtained at the depth of 0.3 mm, where fat tissue (which appears in white since the H&E staining cannot perfuse through hydrophobic tissues) can be distinguished from muscle tissue (which is represented in pink due to the eosin staining). The slice in Figure 4c is representative of the whole sample, since slices at depths higher than 0.3 mm presented similar histological features (slices at depths lower than 0.3 mm were very fibrous and crumbly, thus not suitable for staining).

**Figure 5:**
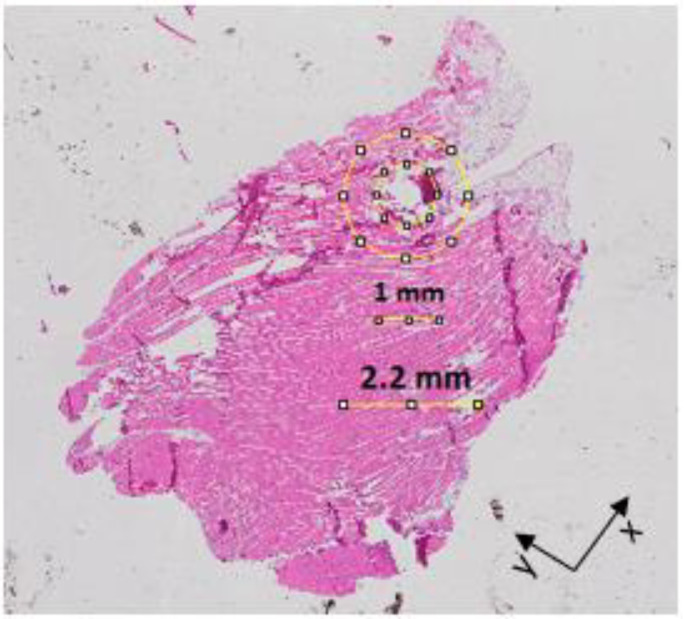
A 5 μm thick histological slice of Sample PM1. On the histological slice, the change in size of the small fat region is highlighted.

Figure 4c also reports the size of the sample, which is 11 mm long and 7.5 mm wide. These dimensions are comparable with the dimensions reported in Table 1, thus suggesting that the sample was appropriately sliced (i.e., that the correct orientation was maintained through sample embedding and slicing). However, although less than 8% difference in size was found for this sample between prefixation and postprocessing (as reported in Table 1), a shrinkage above 50% was found for fat tissue. In fact, while the small fat region had a diameter of approximately 2.2 mm (which is the diameter of the Keysight slim form probe) before fixation, a diameter of 1 mm was measured for the same region after sample processing. An imaged slice for the small fat region is highlighted and compared with the prefixation size in Figure 5.

**Figure 6:**
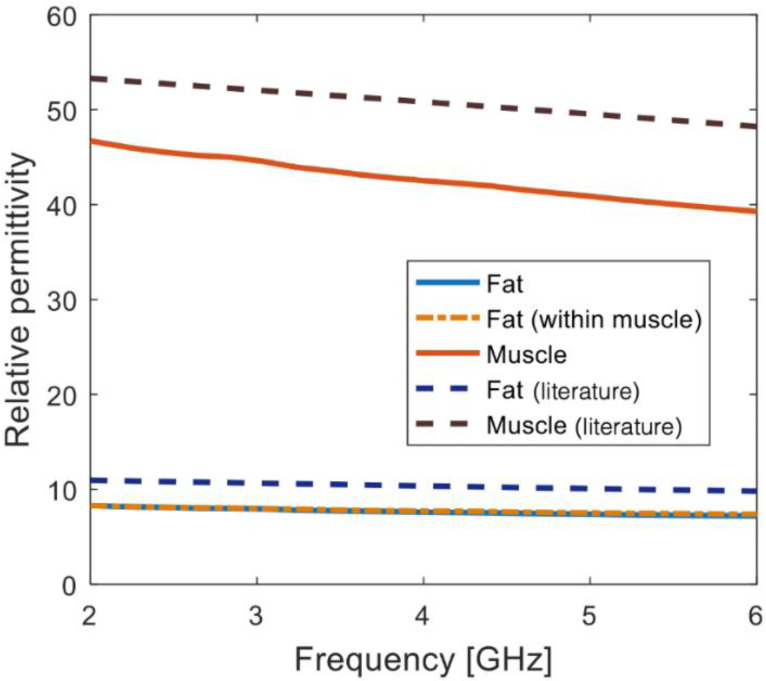
Relative permittivity traces from three measurements performed on Sample PM1. Among the three measurements, one measurement was performed on muscle, one measurement on a larger fat region (approximately twice larger than the probe) and one measurement on a smaller fat region (approximately as large as the probe). Data from measurements on muscle and fat reported in the literature [24] are also plotted with dashed lines. Conductivity traces are not reported since trends similar to relative permittivity were found across the three measurements.

The fat tissue shrinkage highlighted in Figure 5 suggests that the sensing radius estimated in [19] for fat, i.e., 0.9 mm, cannot be used to define the histology region. In fact, if a histology region with a radius of 0.9 mm (i.e., a diameter of 1.8 mm) was considered for the measurement site highlighted in Figure 5, the histology region would include 0.5 mm of fat surrounded by muscle. However, the true histology region should actually include only fat, as suggested by the dielectric traces in Figure 6, which are from three measurements performed on Sample PM1. Specifically, the three dielectric traces in Figure 6 are from one measurement on muscle, one measurement on a larger fat region (approximately twice larger than the probe) and one measurement on a smaller fat region (approximately as large as the probe). In Figure 6, the muscle and fat reference data from [24] are also plotted.

Differences between measured and reference dielectric data can be attributed to biological variability between and within animals, source regions of the body for fat and muscle, source species, and temperature and handling variations [4,8]. Furthermore, the data from the smaller homogeneous fat region (within muscle) are comparable with the data from the larger homogeneous fat region. In fact, less than 1% difference was found between the dielectric traces of the large and small regions. This difference is well within the measurement uncertainty, indicating that the muscle surrounding the small fat region did not have any impact on the dielectric measurement. In other words, the sensing radius of the probe was less than the size of the small fat region. Thus, the measurements of Sample PM1 confirm the sensing radius findings in [19,20]. Specifically, in [19,20], the sensing radius was calculated numerically to be 0.9 mm when the tissue under the probe is fat. The results here agree with this sensing radius, since the edge of the small region is not detected and, therefore, the sensing radius must be smaller than the extent of the measurement region, i.e., smaller than 1.1 mm.

However, the histology information from the slice in Figure 5 can support the interpretation of the dielectric measurement from the smaller fat region only if the histology region is scaled down by 50% (due to the 50% fat shrinkage). In fact, by scaling down the sensing radius of 0.9 mm by 50%, a sensing radius of 0.45 mm is found. Such a value is within the 0.5 mm radius fat region, thus confirming that only fat is included within the histology region illustrated in Figure 5.

Furthermore, inconsistent fat shrinkage of up to 90% was found for the remaining measurement sites of Sample PM1, thus compromising the interpretation of the dielectric data measured from the concentrically heterogeneous measurement sites. Similar results were found for all ovine and porcine muscle samples. Thus, due to the irreversible and unpredictable shrinkage of fat tissue caused by the histology process, the sensing radius and dielectric contribution findings in [17,19,20] could not be directly applied to define the histology region. For this reason, the histology process could not support the interpretation of the dielectric data measured from ovine and porcine muscle and fat samples.

**Figure 7:**
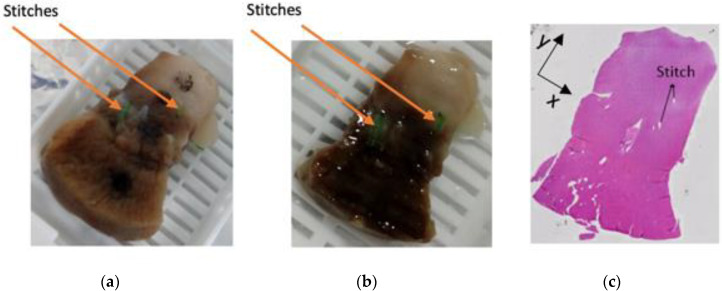
(**a**) Postfixation picture, (**b**) postprocessing picture and (**c**) 10 μm histological slice of Sample LK2 (reported in Table 1). Among the four marked measurement sites in (**a**), two are from the medulla, two from the cortex (with one site closer to the medulla than the other) and one is at the cortex–medulla interface. The two measurement sites at/closer to the interface were further marked with green stitches, since the black marks faded away during sample processing, as is clear in (**b**).

On the other hand, the histology process was able to enhance the interpretation of dielectric data measured from a subset of ovine kidney samples. As an example, Figure 7 shows the histology of Sample LK2 (previously reported in Table 1 and illustrated in Figure 2). In order to facilitate the association of the dielectric data with the histological tissue content, Figure 7 reports postfixation and postprocessing sample pictures together with a sample slice imaged with the microscope. Figure 7a presents four marked measurement sites: one on the medulla, one at the interface between medulla and cortex, and two on the cortex (one close to the interface and one far from it). The measurement sites at and closer to the interface were further marked with green stitches, since the black marks faded away during sample processing, as is clear from Figure 7b. Furthermore, Figure 7c reports the 10 μm slice obtained at the depth of 0.5 mm. A 10 μm instead of a 5 μm slice is reported since it enables distinction between the medulla and cortex because of the additional cell layer present in the 10 μm slice compared to the 5 μm slice. The slice in Figure 7c is representative of the whole sample. Slices at depths higher than 0.5 mm presented the same histological features, thus suggesting that the sample was heterogeneous only radially (slices at depths lower than 0.5 mm were very fibrous and crumbly, thus not suitable for staining, and not analysed further). 

**Figure 8:**
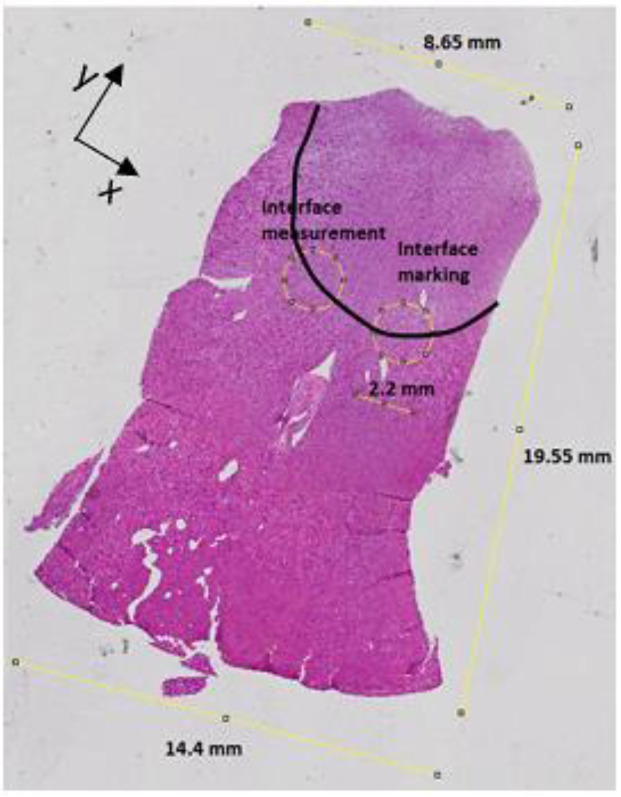
10 μm histological slice of Sample LK2 (reported in Table 1). On the histological slice, the estimated location of the medulla and the histology region at the cortex–medulla interface (marked with the stitch) are highlighted, in order to facilitate the correspondence between dielectric data and histological information.

On the histological slice in Figure 7c, the extremities of the green stitch marking the measurement site at the interface between the cortex and medulla are highlighted, since this site is used to define the histology region and estimate the dielectric contribution of the two side-by-side tissues (i.e., medulla and cortex). In order to enhance the interpretation of the dielectric data from the cortex–medulla interface, the medulla and the histology region at the cortex–medulla interface (marked with the stitch) are highlighted on the 10 μm histological slice in Figure 8. Specifically, the histology region was identified by considering the location of the probe (which has diameter of 2.2 mm) and the distance of the measurement site from the stitch.

**Figure 9:**
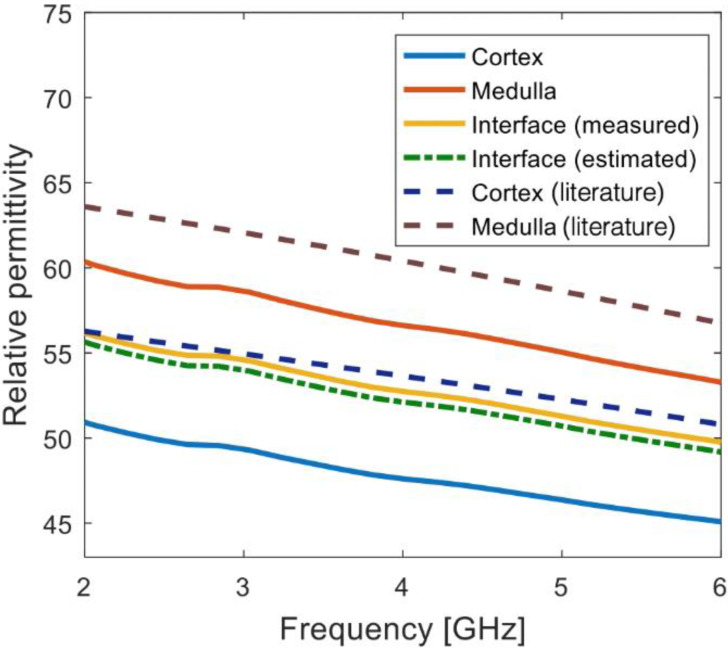
Relative permittivity traces from three measurements performed on Sample LK2. Among the three measurements, one measurement was performed on the medulla, one measurement on the cortex (far from the medulla) and one measurement at the cortex–medulla interface. Data from measurements on cortex and medulla, reported previously by our group [7], are also plotted with dashed lines. Conductivity traces are not reported since trends similar to relative permittivity were found across the three measurements.

Furthermore, Figure 8 reports the dimensions of the slice as a comparison with the dimensions of Sample LK2 reported in Table 1. The dimensions in Figure 8 are the same as the dimensions in Table 1, thus suggesting that the sample was appropriately sliced in the desired orientation. The dielectric traces of Sample LK2 corresponding to the measurements on the cortex, medulla and cortex–medulla interface are reported in Figure 9. For an enhanced interpretation of the dielectric data, Figure 9 reports the reference data from [7] and the estimated signal at the interface obtained by averaging the cortex and medulla signals. The estimated signal from the average properties is the expected trace based on the finding that side-by-side tissues contribute equally to a dielectric measurement, when the probe is centred on the interface between the tissues, as presented in [17,19,25].

The measured cortex and medulla data differed from the reference data from [7] by up to 10%. Such differences can be mainly attributed to tissue dehydration, as seen by the fact that the data from the medulla (i.e., the inner layer of kidney) are closer to the reference data than the data from the cortex (i.e., the outer layer of kidney), which tend to dehydrate more rapidly than the medulla. However, measuring the exact magnitude of the properties is not the aim of this study, as the actual properties are not as important as comparing the tissue dielectric contributions.

Furthermore, in Figure 9, the data from the cortex–medulla interface are comparable with the estimated data obtained by averaging the data from the cortex and medulla. In fact, less than 1% difference was found between the two dielectric traces, thus suggesting that the cortex and medulla had equal dielectric contribution to the dielectric signal acquired at the interface. Thus, with the support of the histological information summarised in Figure 8, the measurements of Sample LK2 reported in Figure 9 confirmed the dielectric contribution findings regarding side-by-side tissues in [17,19]. Similarly, the histological process was able to support the dielectric interpretation of the dielectric data measured from the remaining ovine kidney samples. The key outcomes from the histological analysis conducted across all investigated samples are summarised and compared with the findings from [17,19,20,25] in Table 2.

Overall, the results of this investigation suggest that inconsistencies in the reported dielectric data of heterogeneous tissues are likely caused by errors or weaknesses in linking histological results with dielectric measurements, or in other words, in interpreting the dielectric measurements based on the histological results. This interpretation can be error-prone, even when the dielectric measurement is accurate and the histological process is performed correctly.

## 4. Conclusions

In this work, heterogeneous tissue samples were analysed histologically after measuring their dielectric properties. In particular, the challenges of conducting post-measurement histological analysis were investigated with the aim of supporting accurate dielectric characterisation of heterogeneous tissue samples.

This study found that the sensing radius and dielectric contribution findings from [17,19,20] on samples that did not undergo histology, could be directly applied to define the histology region within only a small subset of radially heterogeneous samples (ovine kidney samples). In this way, ovine kidney samples were accurately characterised through histology. However, the post-measurement histological process was destructive for the remaining samples (porcine and ovine muscle samples) and reliable identification of the histology region was not possible. For instance, the sample processing caused a tissue shrinkage of more than 50% in porcine and ovine muscle samples. For a subset of these samples, the tissue shrinkage could be monitored and taken into account when defining the histology region. However, for the majority of muscle samples, the shrinkage was difficult to monitor and led to the inapplicability of the sensing radius and dielectric contribution findings from [17,19,20].

These results suggest that histology can cause different changes in tissue morphology depending on the tissue constituents in the sample, and importantly, that these changes in tissue morphology can lead to erroneous definition of the histology region, and thus erroneous interpretation of the measured dielectric properties. Such limitations of the post-measurement histology process have not been taken into account in dielectric studies to date and may be the reason why dielectric data from heterogeneous biological samples have been inconsistent in the literature. 

In summary, this study demonstrated that, due to the invasive and destructive process of histology, post-measurement histological analysis is not always able to support the dielectric characterisation of heterogeneous tissue samples. In future studies, methods and reporting standards should be devised for measuring tissue deformation and taking it into account when using histology for dielectric tissue characterisation. In other fields, for example those in which it is required to measure the size of tumours for disease staging, corrections due to tissue shrinkage through the histological process have been investigated [26], and researchers in the field of dielectric measurements may be able to learn from and build upon such efforts. 

Another interesting option for future work is the potential use of mixture theory to correlate the volume fraction of a specific type of cell present in the sample with the measured dielectric properties. In [14], Bruggeman’s effective medium approximation theory was used to describe a heterogeneous tissue sample as a mixture of cells, with each type of cell having different dielectric properties. More work is needed to identify the best type of mixture model to use, and to determine the accuracy of such a model in associating histological imaging results with measured dielectric data. 

Alternatively, future studies could evaluate the use of less invasive and nondestructive techniques, such as microCT [27,28], to support the correspondence between the measured dielectric data and the tissue constituents of heterogeneous samples. However, like histology, microCT is expensive and requires specialist knowledge. Future work should investigate other potential methods that can satisfy the requirements of being nondestructive for tissue samples, while also being readily available and usable by researchers who conduct dielectric measurements. 

Overall, better methods to associate the tissue content with dielectric measurements are needed. Obtaining a solid understanding of the dielectric properties of heterogeneous tissues, in turn, will support the design and development of electromagnetic diagnostic and therapeutic devices.

## Figures and Tables

**Table 1:** Change in size for a subset of radially heterogeneous samples during histology, from prefixation to postprocessing. The sizes refer to cross-sections of the sample through the longitudinal (xy) plane with the bases or bottom/top bases parallel to the x axis and the height parallel to the y axis. All samples were fixed for three days, except for Sample PM3 which was fixed for two days.

Sample (Shape)	Prefixation Size [mm]	Postfixation Size [mm]	Postprocessing Size [mm]
LK1 (triangular)	Base: 15.12; Height: 16.75	Base: 15.21; Height: 16.05	Base: 15.62; Height: 12.68
LK2 (trapezoidal)	Bottom base: 13.84; Top base: 9.85; Height: 24.44	Bottom base: 16.02; Top base: 9.50; Height: 26.37	Bottom base: 14.39 Top base: 8.65; Height: 19.54
PM1 (rectangular)	Base: 9.68; Height: 8.66	Base: 11.64; Height: 7.62	Base: 10.17; Height: 6.52
PM2 (rectangular)	Base: 18.43; Height: 13.42	Base: 19.17; Height: 14.44	Base: 15.19; Height: 13.34
PM3 (rectangular)	Base: 16.87; Height: 13.24	Base: 15.76; Height: 15.37	Base: 13.12; Height: 11.35

**Table 2:** Comparison of the key outcomes from this study (on samples analysed after post-measurement histology) with the outcomes from the literature (on unprocessed tissue samples) [17,19,20,25].

Scenario	Literature (Unprocessed Samples)	This Study (Samples Processed with Histology)
Sensing radius estimation	Sensing radius was 0.9 mm for fat [19]	Histology radius was 0.45 mm for fat (due to ~50% fat shrinkage)
Dielectric contribution of concentric tissues	The contribution of the inner tissue is dominant relative to that of the outer tissue [17,19,20]	Histology caused changes in the morphology of the samples and could not support the literature outcome
Dielectric contribution of side-by-side tissues	Two side-by-side tissues have equal dielectric contribution when probe placed on interface [17,19,25]	Histology supported the literature outcome
